# Characterisation and analysis of thioredoxin peroxidase as a potential antigen for the serodiagnosis of sarcoptic mange in rabbits by dot-ELISA

**DOI:** 10.1186/1471-2334-13-336

**Published:** 2013-07-22

**Authors:** Runhui Zhang, Wanpeng Zheng, Xuhang Wu, Quwu Jise, Yongjun Ren, Xiang Nong, Xiaobin Gu, Shuxian Wang, Xuerong Peng, Songjia Lai, Guangyou Yang

**Affiliations:** 1 Department of Parasitology, College of Veterinary Medicine, Sichuan Agricultural University, Ya’an 625014, China; 2 Sichuan Academy of Animal Husbandry Sciences, Chengdu 610066, China; 3 Department of Chemistry, College of Life and Basic Science, Sichuan Agricultural University, Ya’an 625014, China; 4 College of Animal Science and Technology, Sichuan Agricultural University, Ya’an 625014, China

**Keywords:** *Sarcoptes scabiei*, Thioredoxin peroxidase, Immunolocalisation, Dot-ELISA, Serodiagnosis

## Abstract

**Background:**

Scabies caused by *Sarcoptes scabiei* is a widespread but a neglected tropical zoonosis. In this study, we characterised a *S*. s*cabiei* thioredoxin peroxidase (SsTPx) and evaluated a recombinant SsTPx as a diagnostic antigen in rabbits.

**Methods:**

The open reading frame of the gene encoding SsTPx-2 was amplified and the recombinant protein was expressed in *Escherichia coli* cells and purified. SsTPx was localized in mite tissue by immunolocalisation using the purified recombinant protein. Serodiagnosis assays were carried out in 203 New Zealand White rabbit serum samples by dot-ELISA.

**Result:**

The open reading frame (489 bp) of the gene encodes an 18.11 kDa protein, which showed highly homology to that of *Psoroptes cuniculi* (98.77% identity) and belongs to the 2-Cys family of peroxiredoxins. SsTPx was mainly distributed in muscle tissues of mites, integument of the epidermis and the anterior end of *S*. *scabiei*. Although SsTPx cross-reactivity with psoroptic mites was observed, the SsTPx dot-ELISA showed excellent diagnostic ability, with 95.3% sensitivity and 93.8% specificity in mange-infected and uninfected groups.

**Conclusions:**

This study showed that the purified SsTPx is a highly sensitive antigen for the diagnosis of mange infection by dot-ELISA. This technique is a rapid and convenient method that can be used worldwide for the clinical diagnosis of sarcoptic mange in rabbits, and is especially useful in developing regions.

## Background

*Sarcoptes scabiei* is an ectoparastic mite infecting both humans and animals. Scabies has been reported as a widespread but a neglected tropical disease that is highly contagious in conditions of overcrowding, poverty and poor hygiene [1-3]. More than 100 species of mammals such as companion pets, livestock and wildlife are generally affected, causing severe mortality resulting from the uncontrolled spread of *Sarcoptes *[4] with significant impacts in terms of welfare and economic losses [5-7].

Scabies typically causes pruritus which is frequently more intense at night, and skin inflammation, inducing papular or vesicular lesions related to the burrowing mites and their products [3,7]. Papules and vesicles often develop into excoriations, eczematisations, secondary infections and scabs [8]. However, scabies can be difficult to identify because of severe scratching, inflammation or secondary bacterial infection resulting in misdiagnosis [9]. The pathognomonic evidence for the diagnosis of scabies is the burrow. However, these burrows tend to be invisible to the naked eye so that it should be diagnosed by skilled doctors using advanced techniques such as dermoscopy [10]. A clinical algorithm relevant to itching and lesions, in addition to host biomarkers, are used to differentiate between the stages of scabies and mange [11,12]. An interesting study has demonstrated the use of trained disease-detector dogs that had an efficient capability to detect sarcoptic mange in wildlife [13]. In addition, several reports have described the serodiagnostic methods for scabies and mange. For example, an antigen that is homologous to the house dust mite is a candidate antigen for the diagnosis of sarcoptic mange by immunoscreening [14,15]. *S*. *scabiei* extracts have been used to detect sarcoptic mange infections using enzyme-linked immunosorbent assays (ELISAs) [16-18], but some assays lack appropriate levels of specificity and sensitivity [19]. Thus, the development of efficient methods for the correct identification of scabies and sarcoptic mange is required to reduce the spread of this infection.

Thioredoxin peroxidase (TPx) is a member of peroxiredoxin family (Prx), which is an antioxidant that functions as a peroxidase only when coupled to a sulfhydryl reducing system [20]. Prx has been crucially implicated in protecting organisms from the potentially damaging effects of reactive oxygen species (ROS) and host-activated leukocytes in many parasites [21]. *Opisthorchis viverrini* TPx contributes to the protection of the parasite against damage induced by ROS produced during inflammation [22]. Moreover, thioredoxin peroxidases are widely used in methods for the diagnosis of parasitic diseases. For example, purified recombinant TPx of *E*. *granulosus* was used to screen sera from mice and patients with severe hydatid infections [23]. Furthermore, TPx is considered to be a candidate antigen for the detection of *Schistosoma japonicum* and *Fasciola gigantica* infections in water buffalo [24,25].

The dot-enzyme-linked immunosorbent assay (dot-ELISA) is a simple, rapid and reliable method for screening large number of serum samples [26]. The use of extracts of *Sarcoptes* mites as capture antigens in dot-ELISAs has been established for the diagnosis of sarcoptic mange in rabbits [27]. The aim of this study was to develop a dot-ELISA assay for the serodiagnosis of sarcoptic mange using recombinant TPx protein and to perform the characterisation and immunolocalisation of thioredoxin peroxidase in *Sarcoptes scabiei* (SsTPx).

## Methods

### Mites and samples

Sarcoptic mites (adults, nymphs and larvae) were collected from rabbits and stored at −70°C prior to RNA extraction. The mites were unfed before the start of the experiment to avoid any contamination of the host RNA and proteins. Serum samples were collected from naïve adult New Zealand White rabbits as well as those that had been naturally and experimentally infected with different levels of mites. All animals were handled in strict accordance with the animal protection laws of the People's Republic of China (a draft of an animal protection law in China was released on September 18, 2009). All procedures were carried out in strict accordance with the Guide for the Care and Use of Laboratory Animals by the Animal Ethics Committee of Sichuan Agricultural University (Ya’an, China) (Approval No. 2011–028).

### Cloning, expression and purification of recombinant SsTpx

Total RNA was extracted using a commercial kit (Waston, Shanghai, China) and cDNA was transcribed using RevertAi™ First Strand cDNA Synthesis Kit (Fermentas) according to the manufacturers’ protocols and stored at −70°C. The sequence encoding an open reading frame (ORF) of SsTPx was amplified from the *S*. *scabiei* EST database [28] and *Psoroptes ovis* thioredoxin peroxidase gene using the primers 5’-ccgcaattcATGGCAGTGAAGAATCCG-3’ and 5’-cccaagcttTCAAACTGATCGGCCGAC-3’ (Invitrogen), which incorporated *Eco*RI and *Hin*dIII restriction sites, respectively. The PCR products were digested and gel-purified. The cDNA was subcloned into the bacterial expression vector pET-32a (+) (Novagen, Germany) and used to transform BL21 (DE3) *Escherichia coli* cells (Novagen). *E*.*coli* cells were cultivated in LB medium containing 50 μg/mL ampicillin at 37°C overnight until the OD_600nm_value reached 1.0. Isopropyl-beta-d-thiogalactopyranoside (IPTG) was then added at the final concentration of 1 mM and cells were incubated for a further 4 h at 37°C to induce recombinant SsTPx expression. The purity of the expressed protein was measured as previously described [29].

### Sequence analysis

The presence of a signal peptide was detected using SignalP-4.1 at the Center of Biological Sequence Analysis (http://www.cbs.dtu.dk/services/SignalP-4.1/), and cellular localization was predicted using TMHMM (http://www.cbs.dtu.dk/services/TMHMM/). The molecular weight of the predicted protein was calculated using Compute pI/Mw (http://web.expasy.org/protparam/).

### Western blot analysis

Recombinant SsTPx was separated by SDS-PAGE and transferred onto PVDF membranes (Millipore, Germany) for 1 h in an electrophoretic transfer cell (Bio-Rad, USA). The membrane was blocked with 5% skimmed milk in TBST (40 mM Tris–HCl, 0.5 M NaCl, 0.1 Tween-20, pH 7.4) for 2 h at room temperature. Membranes were then incubated with rabbit antiserum (diluted 1:200 (v/v) in 1% skimmed milk in TBST) overnight at 4°C. After washing with TBST (3×5 min) the membrane was incubated with horseradish peroxidase (HRP)-conjugated goat anti-rabbit antibody for 1 h. After the membrane was washed again with TBST (3×5 min), protein signals were detected using diaminobenzidine (DAB) reagent (Tiangen, China) following the manufacturer’s instructions.

### Immunohistochemistry

In order to perform immunolocalisation studies of mite sections, antiserum against SsTPx was raised in rabbits using standard procedures [30]. Mites were fixed in 1% molten agarose, embedded in paraffin wax after solidification of the molten agarose and cut into sections (5 μm) with a microtome. The sections were dewaxed, rehydrated, treated to inactivate endogenous peroxidase and incubated in 25% normal goat serum in TBS for 15 min. Tissue sections were then incubated with specific rabbit anti-SsTPx antibodies (diluted 1:1000 (v/v) in TBS) overnight at 4°C. After washing three times with TBS the sections were then incubated with horseradish peroxidase (HRP)-conjugated goat anti-rabbit IgG (AMRESCO, Texas, USA) diluted 1:200 for 1 h. Sections were rinsed with TBS then immunoreactivity was detected using the EnVisionTM+ System, HRP (DAB) (DAKO, Glostrup, Denmark). The sections were counterstained with hematoxylin and dehydrated and cleared in xylene. Finally, sections were mounted and viewed with a microscope. Negative controls were prepared using naïve rabbit serum instead of specific antibodies.

### Dot-ELISA

The concentration of coating protein and serum dilution was optimized for use in the SsTPx dot-ELISA. Antigen (2 μg/mL, 6 μg/mL and 24 μg/mL) was dotted onto the nitrocellulose membrane (NCM), which were hydrated with phosphate-buffered saline (PBS; 0.1 M, pH 7.4), for 1 h at room temperature. The membrane was then dried at 37°C for 2 h and cut into strips (approximately 0.5×3 cm), each containing 1 μL antigen. The strips were blocked with PBST containing 5% skimmed milk for 30 min and washed with PBST (PBS, 0.3% Tween-20) three times. Sera (diluted 1:200) were then added and incubated at 37°C for 1 h before being washed with PBST three times. Each strip was then incubated with HRP-conjugated goat anti-rabbit IgG diluted 1:1000 in PBS. Dot-ELISA was developed with the EnVisionTM+ System, HRP (DAB). Serum samples infested with *Psoroptes cuniculi* (n = 12), *Eimeria stiedae* (n = 6) and *Taenia pisiformis* (n = 6) were used as controls for the detection of cross-reactivity. All the dot strips were kept at 4°C. Eighty-five serum samples from infected rabbits were tested and 94 negative controls were treated with naïve rabbit serum. The stability of the strips was also evaluated by detection of infected serum and negative serum samples (n = 6 each) three times.

Results were read on the basis of color variation in the PVDF membranes: brown dots indicated a positive result, while no dots indicated a negative result. Test sensitivity was calculated as: sensitivity = ELISA positive/true positive × 100%. Test specificity was calculated as: specificity = ELISA negative/true negative × 100% [31].

### Evaluation and statistical analysis

Evaluation of dot-ELISAs was based on a previously reported method [32] with the following modification: two independent observers read all the dot-ELISA strips on the same day. Observer 1 read all of the strips again two days later to evaluate observer variability. The kappa index (K) and P values were used to evaluate assay results (http://graphpad.com/quickcalcs/kappa1/).

## Results

### Sequence alignment and expression

**Figure 1:**
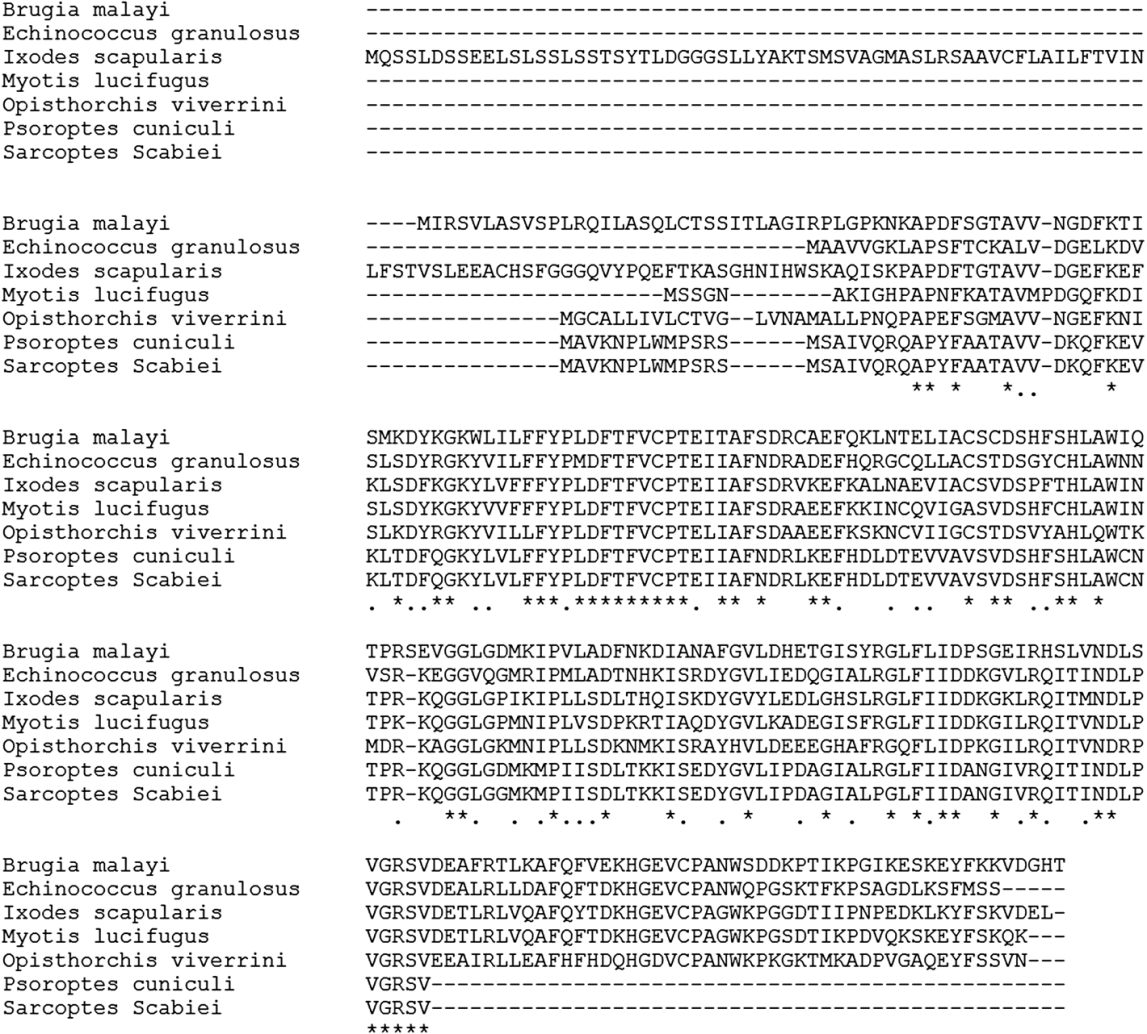
**Amino acid sequence alignment of thioredoxin peroxidase from *Sarcoptes Scabiei *and other species.** GenBank accession numbers: *Brugia malayi*, AAC23701.1; *Echinococcus granulosus*, AAL84833.1; *Ixodes scapularis*, XP_002415327.1; Myotis lucifugus, AAT79401.1; *Opisthorchis viverrini*, ACB13822.1; *Psoroptes cuniculi*, KF241278.

**Figure 2:**
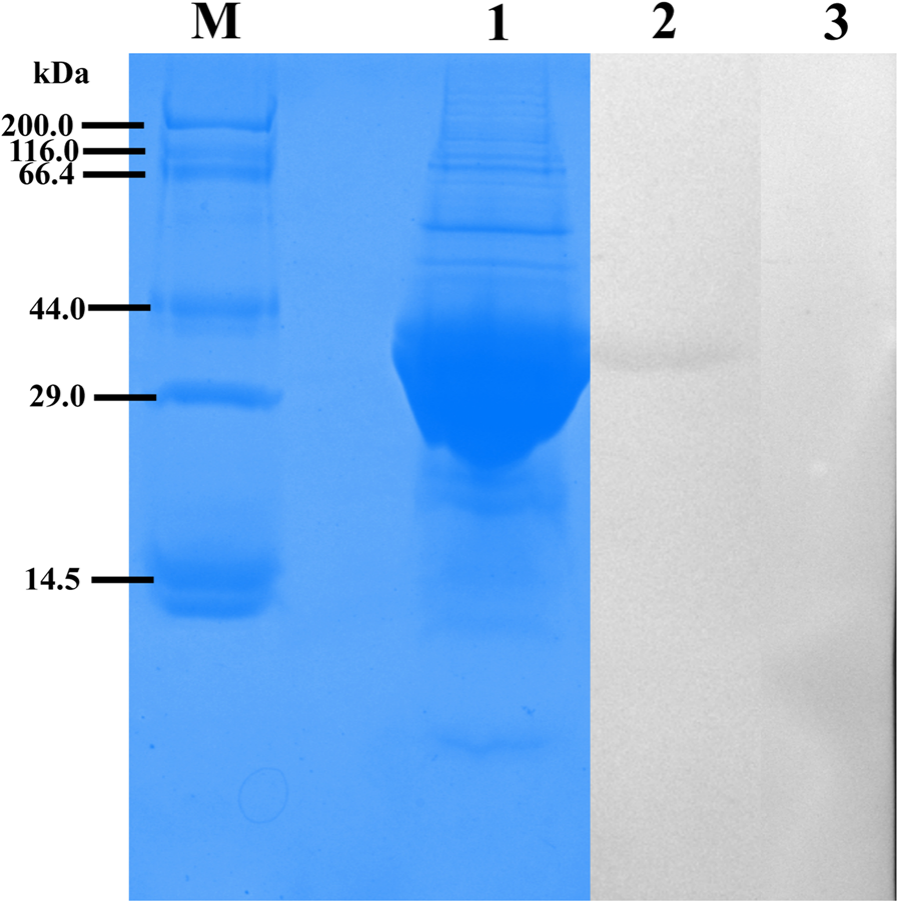
**Western blot analysis of the recombinant SsTPx.** M, protein molecular weight markers; Lane 1, purified recombinant proteins; Lane 2, Western blot detection with *S*. *scabiei* infected rabbit antisera; Lane 3, Western blot detection with naïve serum.

We cloned an ORF of 489 bp encoding a 163 amino acid protein (GeneBank accession number: KC693033). The protein was predicted to have a molecular weight of 18.11 kDa, a pI of 6.82, and weak hydrophobicity. No signal peptides or transmembrane domains were found in this protein. An alignment of SsTPx is shown in Figure 1. SsTPx was found to be highly homologous to that of *Psoroptes ovis* (98.77% identity) and *Psoroptes cuniculi* (98.77% identity) and exhibited homology to that of other parasites such as *Myotis lucifugus*, *Echinococcus granulosus*, *Ixodes scapularis* thioredoxin peroxidase (45.93%, 44.66% and 35.74% identity, respectively). Recombinant SsTPx was significantly expressed as an insoluble protein with a molecular weight of approximately 40 kDa (Figure 2). An additional peptide (20 kDa) was expressed from the pET-32a (+) vector.

### Western blot analysis

Western blot analysis showed that this recombinant protein reacted with *S*. *scabiei* infected rabbit antisera. Immunoreactivity was not observed when negative rabbit serum was used (Figure 2).

### Immunohistochemistry

**Figure 3:**
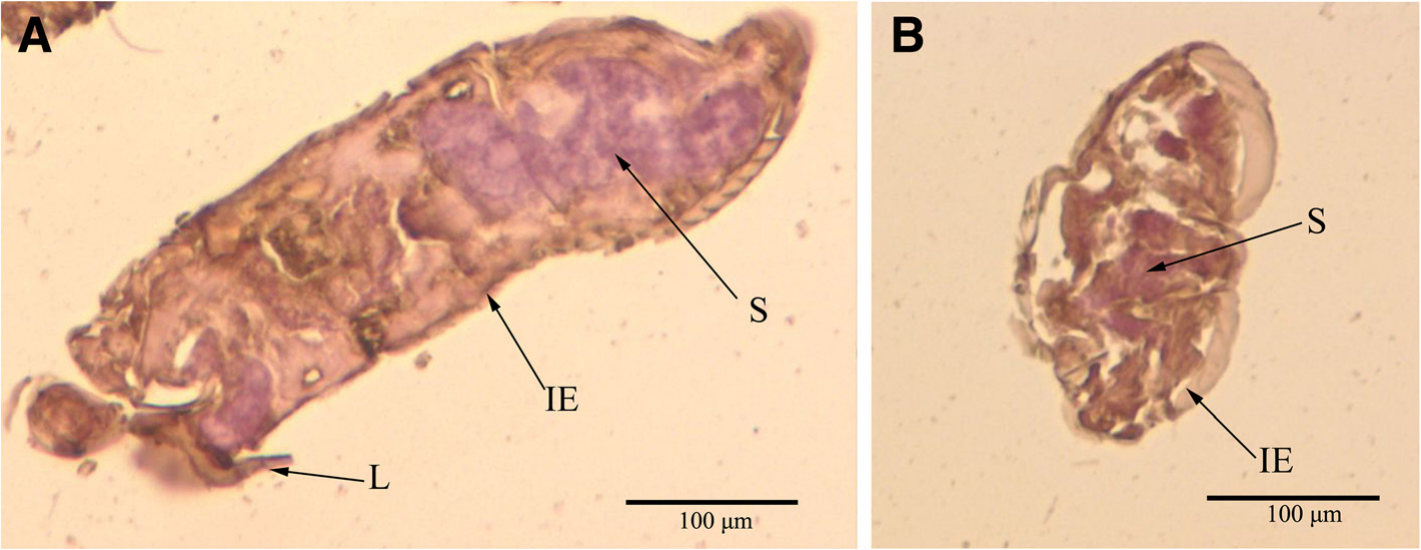
**Immunohistochemistry of SsTPx in sections of *S. scabiei*. (A**, **B)** Staining with SsTPx antiserum as the primary antibody. The EnVision TM + System-HRP(DAB) (DAKO) was used, according to the manufacturer’s instructions for detection of the rabbit antibodies. L, legs; S, stomach blocks; IE, epidermal integument.

Obvious staining was widespread in mite muscle tissue and the epidermal integument. This protein was mainly located in the anterior end of *S*. *scabiei* (Figure 3). No staining was detected with pre-immune serum (data not shown).

### Dot-ELISA

**Figure 4:**
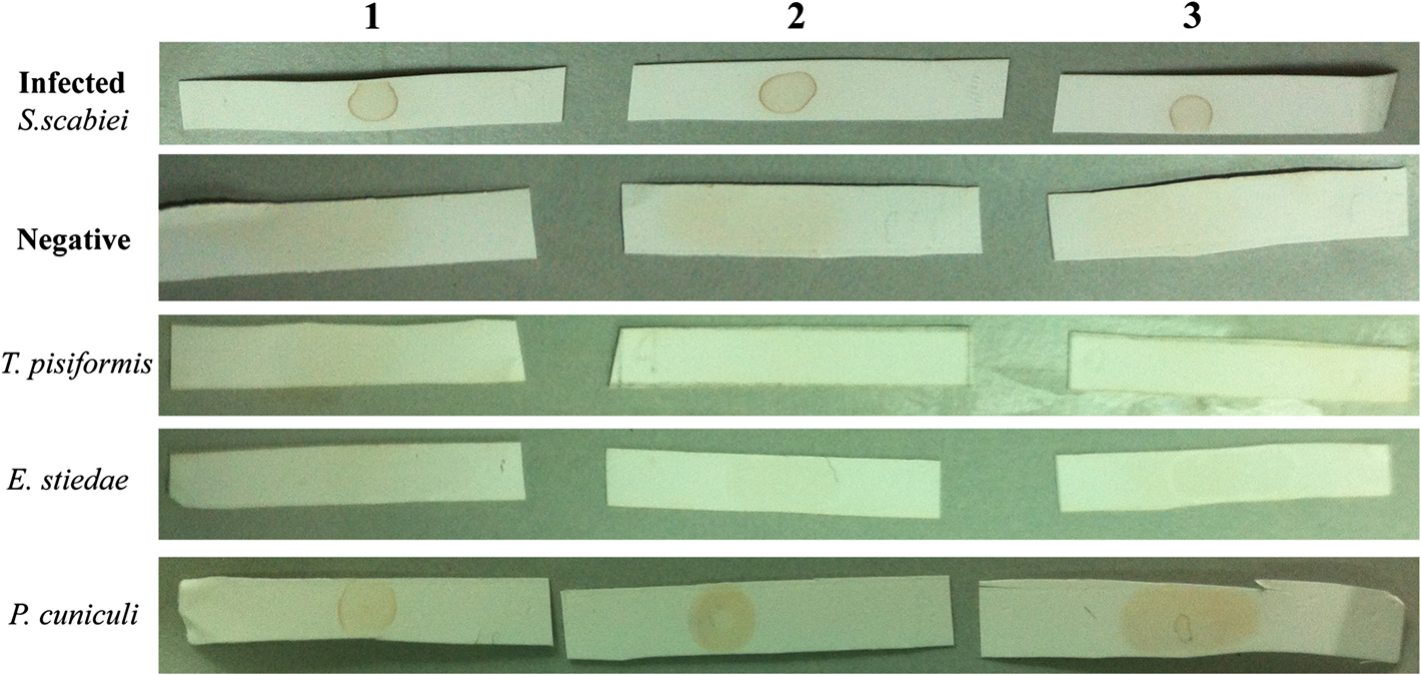
**Dot-ELISA showing reactivity of infected group and uninfected groups using three concentrations of recombinant SsTPx protein as a capture antigen.** Lane 1, 2 μg/mL protein; Lane 2, 6 μg/mL protein; Lane 3, 24 μg/mL protein.

The optimal dilution of the serum and controls for use in dot-ELISA assays was 1:200 and the optimal concentration of the recombinant SsTPx antigen was 2 μg/mL (data not shown). A total of 203 rabbit sera were evaluated. Most experimentally or naturally infected *S*. *scabiei* sera (average 81/85, 95.3% sensitivity) showed positive reactivity in dot-ELISAs, while most *S*. *scabiei*-negative sera (average 111/118, 93.8% specificity) showed no reaction with SsTPx. In the control groups, SsTPx showed no cross-reaction with *E*. *stiedae* (0/6) and *T*. *pisiformis* (0/6). Cross-reactivity was observed with *P*. *cuniculi*-infected (7–8/12) serum although the definition of the dots was less clear that those of the *S*. *scabiei* infected group (Figure 4). No obvious variation was observed among the results for the three time-points tested indicating the stability of the membrane-bound antigen.

The evaluations made by the two observers are shown in Table 1. Among the first evaluations of the *S*. *scabiei* infected group, Observers 1 and 2 reported 95.3% and 94.1% sensitivity, respectively, with a corresponding K index of 0.882 (98% proportion agreement (PA), *P* < 0.01). In the negative and control groups (*E*. *stiedae* and *T*. *pisiformis*), specificity ranged from 98.9% to 100% with K concordance indexes of 0.794, 1.0 and 1.0 (*P* < 0.01), and PA of 98%, 100% and 100%, respectively. Specificity of the *P*. *cuniculi* group was 68.3% (Observer 1) and 66.7% (Observer 2) with a K concordance index of 0.823 (*P* < 0.01) and PA of 91%. The PA between the first and second readings made by Observer 1 was 98% (K = 0.851, *P* < 0.01) in the *S*. *scabiei* infected group (Table 2).

**Table 1:** **Detection of recombinant SsTPx in infected *S***. ***scabiei *serum**, **negative serum and controls by dot**-**ELISA**

**Groups (n = 203)**	**Observer 1 number**		**Sensitivity or specificity**	**Observer 2 number**		**Sensitivity or specificity**	Kappa index*	**Proportion agreement(PA)**
	**+**	**–**		**+**	**–**			
*S*. *scabiei* infected-(n=85)	81	4	95.3%	80	5	94.1%	0.882	98%
Total uninfected (n=118)	7	111	94.1%	8	110	93.2%	0.928	99%
Negative (n=94)	0	94	100%	0	94	100%	1.0	100%
*E*.*stiedae* (n=6)	0	6	100%	0	6	100%	1.0	100%
*T*.*pisiformis* (n=6)	0	6	100%	0	6	100%	1.0	100%
*P*. *cuniculi* (n=12)	7	5	68.3%	8	4	66.7%	0.823	91%

*Strength of agreement: K index <0, poor; 0–0.20, slight; 0.21–0.4, fair; 0.41–0.6, moderate; 0.61–0.80, substantial; 0.81–1.0; almost perfect.

**Table 2:** Kappa index and PA between the first and second readings of observer 1

**Groups (n=203)**	***S*.*scabiei *infected (n=85)**	**Negative (n=94)**	***E*.*stiedae *(n=6)**	***T*.* pisiformis *(n=6)**	***P*. *cuniculi *(n=12)**
Kappa index	0.851	1.0	1.0	1.0	0.833
Proportion agreement(PA)	98%	100%	100%	100%	91%

## Discussion

In the present study, we have described the characterisation and localization of a peroxiredoxin (Prx) from *S*. *scabiei*, and evaluated its use as a diagnosis antigen. The existence of two conserved cysteine residues at positions 61 and 97 demonstrates that this protein belongs to the 2-Cys peroxiredoxin family [33]. 2-Cys TPx in yeast has been shown to have peroxidase activity and intermolecular disulfide linkage [34]. TPx is characterised as an antioxidant enzyme from parasites including *Echinococcus granulosus*, *Fasciola hepatica*, *Schistosoma mansoni* and *Onchocerca volvulus*[35]. The amino acid sequence of SsTPx shows high homology with TPx from *P*. *ovis*. Furthermore, immunohistochemical analysis showed that SsTPx was located in the anterior end of mite, which is similar to the location of TPx from *P*. *ovis*[36]. Also, the detection of the protein in the epidermal integument and muscles close to the epidermis implicates SsTPx in the protection of *S*. *scabiei* against oxidative damage.

Many studies have identified TPx as a candidate antigen for the diagnosis of parasitic infections. Purified TPx has been used as capture antigens for serodiagnosis of *Fasciola gigantica* infection, as well as *Schistosoma japonicum* infection in buffalo [24]. Data have also indicated that the use of specific recombinant TPx from *Echinococcus granulosus* as a capture antigen increased the diagnostic sensitivity of an ELISA for the detection of hydatid infection [37]. In addition, a previous study in our laboratory showed that whole mite extracts can be effectively used as diagnostic antigens by dot-ELISA [27]. ELISA using *S.scabiei* extracts has been used for the diagnosis of sarcoptes mange in animals [16,19]. However, this approach is limited by the lack of an in vitro culture system for mites and and the host cross-reactivity [5]. In comparison to standard plate ELISAs, the dot-ELISA is a simple and accurate method that does not require any specialized equipment. Therefore, this study aimed to evaluate the potential use of recombinant antigens in the diagnosis of sarcoptic mange in rabbits by dot-ELISA.

In our study, sera from rabbits with common parasitic infections were selected as control groups for the detection of cross-reactivity. The lower specificity observed in the *P*. *cuniculi* group may indicate cross-reactivity with *P*. *cuniculi* infection. Infection of rabbits with the zooparasite *P*. *cuniculi* causes serious pruritus and scabs which can completely cover the external ear canal and the internal surfaces of the pinna [38]. However, psoroptic mites, which are significantly larger than scabies mites, are readily visible to the naked eye or otoscope. Moreover, psoroptic mite infection and mange affect different areas of the body in rabbits. Consequently, we considered that the SsTPx antigen was suitable for the detection of sarcoptic mange in rabbits.

Recently, *S.cabiei* recombinant antigens have considered as potential diagnostic tools. A previous study showed that quantification of recombinant allergen-specific IgE levels by DELFIA is highly sensitive method for the diagnosis of scabies infections. This allergen showed excellent diagnostic capability with 100% sensitivity and 93.75% specificity [39]. Recombinant ELISA assay indicated it was a potentially complementary tool for diagnosing swine mange affected by human derived *S*.*scabiei*[40]. In our study, most infected sera were recognized by recombinant SsTPx and no reactivity was observed with negative serum. The assay showed high sensitivity (95.3%) and specificity (93.8%) in three evaluations by two independent observers. Another report showed 92% sensitivity and 96% specificity in serodiagnosis of canine sarcoptic mange by ELISA [41]. The K index revealed predominant concordance between the evaluations of the infected and total uninfected groups made by the two observers as well as that observed between the first and second readings of Observer 1. Thus, the high sensitivity and specificity of the dot-ELISA developed in this study indicate that recombinant SsTPx is a suitable diagnostic antigen. This rapid and convenient diagnostic technique will be of critical importance in the world especially in the developing world where advanced medical instrumentation is unavailable.

## Conclusion

In conclusion, we have characterised a peroxiredoxin from *Sarcoptes scabiei* and analyzed its diagnostic value. Our results demonstrate localization of the protein in muscle tissues and the anterior end of *S*. *scabiei*. The dot-ELISA was developed using purified SsTPx as a capture antigen and exhibited high diagnostic efficiency. This technique represents a rapid and convenient method for the clinical diagnosis of sarcoptic mange in rabbits worldwide especially for the developing regions.

## Competing interests

The authors declare that they have no competing interests.

## Authors’ contributions

RZ participated in the design of the study, manuscript writing and performed the statistical analysis; WZ and JQ participated in experiment and discussion; XW carried out the molecular genetic studies; YR and XN participated in the collection of mites samples and data; XG prepared figures and tables; SW participated in sequence alignment, XP prepared figures and helped to draft the manuscript; SL provided the support of experiment animals and serum samples; GY participated in the design of study and have given final approval of the version; All authors read and approved the final manuscript.

## Pre-publication history

The pre-publication history for this paper can be accessed here:

http://www.biomedcentral.com/1471-2334/13/336/prepub
